# Abstract of Meteorological Observations for August, 1855, Made at Philadelphia, Pa.

**Published:** 1855-10

**Authors:** James A. Kirkpatrick


*Abstract of Meteorological Observations for August,* 1855, *made at Philadelphia, Pa. Latitude* 39° 57′ 28″ *N., Longitude* 75° 10′ 40″ *W. from Greenwich.* By PROF. JAMES A. KIRKPATRICK.

| 1855. August. | BAROMETER. Daily Mean | BAROMETER. Mean Daily Range. | THERMOM. Daily Mean | THERMOM. Mean Daily Range | Dew Point 2 P.M. | Force of Vapor 2 P.M. | Rel. Humid. 2 P.M. | Rain. | Prevailing Winds. | Remarks. |
|---|---|---|---|---|---|---|---|---|---|---|
| | Inches. | Inches. | Deg. | Deg. | Deg. | Inches. | Hunds. | Inch. | Points. | |
| 1 | 29.877 | .078 | 78.3 | 3.3 | 71.5 | .772 | .70 | | E.NE. | Cloudy. |
| 2 | 29.940 | .064 | 75.8 | 2.5 | 66.2 | .644 | .64 | | NE. | Cloudy. |
| 3 | 29.999 | .058 | 76.2 | 0.7 | 72.0 | .783 | .82 | 0.168 | (Var.) | Cloudy; aft. *rain.* [l'ng. |
| 4 | 29.827 | .172 | 77.0 | 3.2 | 72.2 | .788 | .68 | 0.326 | (Var.) | M. & A. cl'r; ev. *rain,* th'r & |
| 5 | 29.695 | .131 | 74 5 | 3.2 | 69.3 | .716 | .77 | 0.254 | NE. | Cloudy; ev. and night *rain.* |
| 6 | 29.740 | .052 | 76.5 | 5.0 | 66.4 | .648 | .59 | | NW. | M. & aft. cloudy; ev. clear. |
| 7 | 29.873 | .133 | 75.8 | 2.0 | 69.6 | .723 | .75 | 0.027 | W. | M. & ev. cl'r; aft. *R.* showers |
| 8 | 29.946 | .084 | 72.0 | 3.8 | 63.1 | .577 | .65 | 0.034 | NE,E,SE. | Cloudy; *rain* during night. |
| 9 | 29.546 | .400 | 78.3 | 6.3 | 75.2 | .872 | .86 | 0.131 | SW. | M. cl'y, A. *r'n,* E. cl'r. *Bar.* |
| 10 | 29.642 | .096 | 75.5 | 2.7 | 56.4 | .455 | .46 | | W. | Clear. [*lowest* 29.514. |
| 11 | 29.922 | .280 | 72.0 | 3.5 | 53.0 | .402 | .45 | | N,NE,E | Clear. |
| 12 | 30.107 | .185 | 73.7 | 1.7 | 61.0 | .537 | .55 | | E. | Clear. |
| 13 | 29.973 | .134 | 79.0 | 5.3 | 71.5 | .772 | .70 | | (Var.) | M. and aft. cl'dy; ev. clear. |
| 14 | 29.971 | .035 | 78.0 | 1.3 | 61.0 | .534 | .49 | | NW,N,NE. | M. and ev. clear; aft. cl'dy. |
| 15 | 29.943 | .037 | 77.5 | 1.2 | 65.3 | .624 | .58 | 0.138 | NE,SE. | Cloudy; ev. *rain.* |
| 16 | 29.766 | .177 | 80.8 | 3.3 | 74.2 | .845 | .75 | 0.014 | SE,SW. | M. *rain,* aft. cl'dy, ev. clear. |
| 17 | 29.743 | .075 | 80.3 | 2.8 | 68.8 | .704 | .60 | | SW. | Cloudy. *Th. highest* 84° |
| 18 | 30.051 | .308 | 70.7 | 9.7 | 47.8 | .332 | .39 | | NW. | Clear. |
| 19 | 30.216 | .166 | 67.7 | 3.0 | 50.6 | .368 | .46 | | N,W,S. | Clear. *Th. lowest,* 55° |
| 20 | 30.229 | .017 | 70.8 | 4.2 | 51.6 | .382 | .44 | | S. | Clear. *Bar. highest* 30.255. |
| 21 | 30.146 | .083 | 73.0 | 2.5 | 59.8 | .514 | .54 | | (Var.) | M. and aft. clear; ev. cl'dy. |
| 22 | 30.036 | .110 | 76.3 | 3 3 | 66.6 | .651 | .66 | | (Var.) | Cloudy. [& lightning. |
| 23 | 29.751 | .285 | 78.8 | 3.8 | 74.2 | .845 | .75 | 1.880 | SSW. | Cloudy; M. & aft. *rain,* th'r |
| 24 | 29.743 | .081 | 79.2 | 1.7 | 71.0 | .758 | .67 | | W. | Cloudy. M. fog. |
| 25 | 29.936 | .193 | 78.5 | 1.5 | 63.4 | .584 | .50 | | NE. | Cloudy. |
| 26 | 29.906 | .055 | 78.3 | 1.7 | 75 2 | .873 | .83 | 0.193 | S,SW,SE. | Cloudy; *rain,* th'r & lt'ng. |
| 27 | 29.968 | .063 | 70.8 | 7.5 | 62.5 | .566 | .73 | 0.072 | NE. | Cloudy. M. *rain.* |
| 28 | 29.990 | .036 | 70.2 | 3.3 | 54.8 | .429 | .51 | | NE. | M. and aft. cl'dy; ev. clear. |
| 29 | 29.921 | .081 | 69.5 | 4.0 | 58.1 | .484 | -55 | | NW,W,SW. | Clear. |
| 30 | 29.977 | .128 | 73.0 | 5.2 | 55.7 | .444 | .46 | | NW. | Clear. |
| 31 | 30.229 | .252 | 67.7 | 5.3 | 56.9 | .463 | .55 | | NE. | M. & ev. clear, aft. cloudy. |
| Means for Aug., 1855 | 29.923 | .131 | 75.0 | 3.5 | 64.0 | .616 | .62 | 3.237 | N. 33½° W. 7-100. | |
| Means for Aug., 5 yrs. | 29.921 | .107 | 74.0 | 4.1 | 58.4 | | | 2,909 | N. 83½° W. 14-100. | |
| Summer Means, 1855 | 29.838 | .114 | 75.5 | 3.9 | 64.0 | .622 | .60 | 17.839 | S. 77° W. 30-100. | |
| Summer Means, 4yrs | 29.898 | .097 | 74.8 | 4.0 | 58.0 | | | 12.939 | S. 76½° W. 25-100. | |

The Monthly Range of the Barometer was 0.741 of an inch, and of the Thermometer 29°.

